# Surveillance of cell wall diffusion barrier integrity modulates water and solute transport in plants

**DOI:** 10.1038/s41598-019-40588-5

**Published:** 2019-03-12

**Authors:** Peng Wang, Monica Calvo-Polanco, Guilhem Reyt, Marie Barberon, Chloe Champeyroux, Véronique Santoni, Christophe Maurel, Rochus B. Franke, Karin Ljung, Ondrej Novak, Niko Geldner, Yann Boursiac, David E. Salt

**Affiliations:** 1 Institute of Biological and Environmental Sciences, University of Aberdeen, Aberdeen, AB24 3UU UK; 2 Biochimie & Physiologie Moléculaire des Plantes, Univ Montpellier, CNRS, INRA, SupAgro, Montpellier, France; 3 Present Address: Division of Plant and Crop Sciences, Future Food Beacon of Excellence & School of Biosciences, University of Nottingham, Nottingham, LE12 5RD UK; 4 Department of Plant Molecular Biology, University of Lausanne, 1015 Lausanne, Switzerland; 5 Department of Ecophysiology, Institute of Cellular and Molecular Botany, University of Bonn, 53115 Bonn, Germany; 6 Umeå Plant Science Centre, Department of Forest Genetics and Plant Physiology, Swedish University of Agricultural Sciences, SE-901 83 Umeå, Sweden; 7 Laboratory of Growth Regulators, Centre of the Region Haná for Biotechnological and Agricultural Research, Faculty of Science of Palacký University & Institute of Experimental Botany of the Czech Academy of Sciences, Olomouc, Czech Republic; 8 Present Address: Department of Agronomy and Horticulture, University of Nebraska Lincoln, Lincoln, NE 68588-0660 USA; 9 Present Address: Department of Botany and Plant Biology, University of Geneva, 30, quai Ernest-Ansermet, CH-1211 Geneva 4, Switzerland

## Abstract

The endodermis is a key cell layer in plant roots that contributes to the controlled uptake of water and mineral nutrients into plants. In order to provide such functionality the endodermal cell wall has specific chemical modifications consisting of lignin bands (Casparian strips) that encircle each cell, and deposition of a waxy-like substance (suberin) between the wall and the plasma membrane. These two extracellular deposits provide control of diffusion enabling the endodermis to direct the movement of water and solutes into and out of the vascular system in roots. Loss of integrity of the Casparian strip-based apoplastic barrier is sensed by the leakage of a small peptide from the stele into the cortex. Here, we report that such sensing of barrier integrity leads to the rebalancing of water and mineral nutrient uptake, compensating for breakage of Casparian strips. This rebalancing involves both a reduction in root hydraulic conductivity driven by deactivation of aquaporins, and downstream limitation of ion leakage through deposition of suberin. These responses in the root are also coupled to a reduction in water demand in the shoot mediated by ABA-dependent stomatal closure.

## Introduction

Both Casparian strips and suberin lamellae, two extracellular hydrophobic barriers located in the wall of endodermal cells of the root, are thought to play important roles in restricting the free diffusion of solutes and water (reviewed in^1,2^). Casparian strips act as apoplastic barriers not only to block solutes moving into the xylem through the free space between cells, but also to prevent their backflow from the stele to the apoplast of the cortex^3–5^. Suberin lamellae, due to their deposition between the endodermal plasma membrane and secondary cell wall, do not block aploplastic transport but rather limit transcellular transport of nutrients^6,7^ and possibly water at the endodermis. Cross talk between the Casparian strip and suberin lamellae exists, with suberin being deposited in response to disruption of Casparian strips^3–5,7,8^. These extracellular barriers are therefore at a cross-road between control of mineral nutrient and water uptake. However, the mechanisms that allow plants to integrate both these barrier functions to enable the simultaneous uptake of sufficient water and mineral nutrients remain underexplored.

The dirigent-like protein Enhanced Suberin1 (ESB1) functions in the correct formation of Casparian strips by allowing the lignin, deposited at the Casparian Strip Domain through the action of Peroxidase64 (PER64) and the Respiratory Burst Oxidase Homolog F (RBOHF)^9^, to form into a continuous ring^3^. In the absence of this dirigent-like protein defective Casparian strips are formed along with enhanced and early deposition of suberin in the endodermis^3^. A similar pattern of Casparian strip disruption and response is also observed when the Casparian Strip Domain (CSD) is disrupted through the loss of Casparian Strip Domain Proteins (CASPs)^3^. These changes lead to systematic alterations in the profile of mineral nutrients and trace elements accumulating in leaves, and this phenotype provided the first tool for identification of genes involved in Casparian strip development^10^. Detection of the diffusible vasculature-derived peptides CASPARIAN STRIP INTEGRITY FACTORS 1 & 2 (CIF1 & 2) through interaction with the SCHENGEN3 receptor-like kinase is what drives this endodermal response to loss of Casparian strip integrity^4,11,12^.

Here, we report that detection of a loss of Casparian strip integrity at the root endodermis by the CIFs/SGN3 pathway leads to an integrated local and long-distance response. This response rebalances water and mineral nutrient uptake, compensating for breakage of the Casparian strip apoplastic seal between the stele and the cortex. This rebalancing involves both a reduction in root hydraulic conductivity driven by deactivation of aquaporins, and limitation of ion leakage through deposition of suberin in endodermal cell walls. This local root-based response is also coupled to a reduction in water demand in the shoot driven by ABA-mediated stomatal closure.

## Results and Discussion

### Loss of Casparian strip integrity leads to enhanced suberin deposition

**Figure 1:**
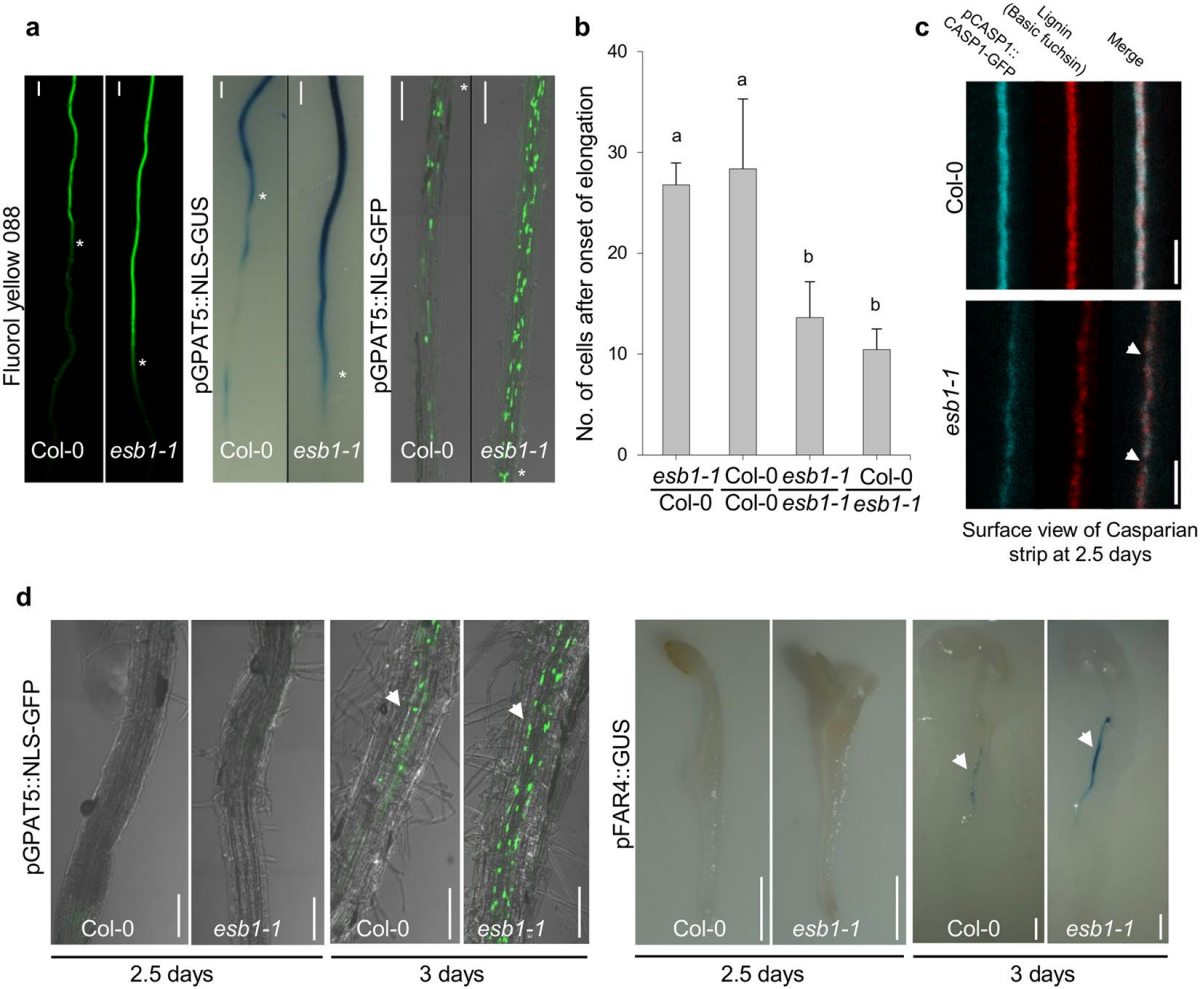
Enhanced endodermal suberin in *esb1-1* is driven by loss-of-function of *ESB1* in roots. (**a**) Suberin staining with Fluorol yellow 088, promoter activity of suberin biosynthetic gene *GPAT5* revealed with GUS staining and nuclear localized GFP (NLS-GFP), (bottom part: toward the root tip; top part: toward the hypocotyl) in 7 day-old seedlings. The white asterisks indicate the beginning of continuous Fluorol yellow 088 staining, GUS expression or GFP in either Col-0 or *esb1-1*. Scale bars: 100 μm. (**b**) Number of endodermal cells from the first fully expanded cell to the beginning of continuous Fluorol Yellow 088 staining in the lateral roots of 2 week-old grafted plants, including *esb1-1* shoot/wild-type Col-0 root grafted, self-grafted wild-type Col-0, self-grafted *esb1-1*, and Col-0 shoot/*esb1-1* root grafted. Different letters indicate the significant differences between means in pairwise comparison by Duncan’s MRT test (α = 0.05), means ± SE, n = 7. Fluorescence of lignin stained with basic fuchsin and pCASP1::CASP1-GFP (**c**) in wild-type Col-0 and *esb1-1* in 2.5 day-old seedlings. The white arrows in (**c**) represent the disrupted lignification/CASP1-GFP localisation in *esb1-1* compared to wild-type Col-0. Scale bars: 5 μm. GFP fluorescence and GUS staining (**d**) in suberin biosynthetic gene promoter reporter lines *pGPAT5::NLS-GFP* and *pFAR4::GUS*, respectively, at 2.5 days and 3 days after sowing in wild-type Col-0 and *esb1*-*1*. The white arrowheads show GFP fluorescence and GUS activity in *esb1*-*1* in 3 day-old plants. Scale bars (**d**, left panel): 100 μm, (**d**, right panel): 1 mm, n = 3 roots were observed each time point and genotype, and representative pictures shown.

The dirigent-like protein Enhanced Suberin1 (ESB1) functions in the formation of Casparian strips by allowing the correct deposition of lignin at the Casparian strip domain. The enhanced deposition of suberin in the *esb1-1* mutant with disrupted Casparian strips can clearly be observed using the lipophilic stain Fluorol Yellow 088 (FY 088) close to the root tip (Fig. 1a), and this can be quantified by counting the number of endodermal cells after the onset of cell expansion to the first appearance of yellow fluorescence (Fig. S1a). This early deposition of suberin is also verified by the clear correspondence of FY 088 staining with enhanced promoter activity of known suberin biosynthetic genes^13^, including *GPAT5* monitored through both GUS staining and GFP fluorescence (Fig. 1a), and also others through GUS staining (*FAR1*, *FAR4*, *FAR5*, *GPAT5*) (Supplementary Fig. S1a,b). This is further reinforced by the enhanced expression of known suberin biosynthetic genes (*FAR1*, *FAR4*, *FAR5*, *GPAT5*, *HORST* and *MYB41*) in *esb1-1* relative to wild-type (Supplementary Fig. S1c).

To better understand the causal link between Casparian strip integrity and enhanced deposition of suberin, we performed a reciprocal grafting experiment that revealed that the *esb1-1* mutation is only required in the root to drive enhanced deposition of suberin at the endodermis, placing the function of ESB1 and the driver for increased suberin in the same tissue (Fig. 1b and Supplementary Fig. S1d). To determine the cause and effect relationship between damaged Casparian strips and enhanced suberin we carefully monitored the first appearance of both Casparian strips and enhanced suberin in *esb1-1*. Using lignin staining in the Casparian strip marker line *pCASP1::CASP1::GFP*, we are able to observe that damaged Casparian strips are visible 2.5 days after sowing (Fig. 1c). This is at least 12 hr before the first indication of enhanced suberin biosynthesis, which we monitor using promoter activity of suberin biosynthetic genes *GPAT5*, *FAR4*, *FAR1* and *FAR5* (Fig. 1d; Supplementary Fig. S2a). This was also verified by the direct observation of suberin deposition with FY 088 (Supplementary Fig. S2b). The observation that treatment with the CIF2 peptide, normally leaked from the stele through loss of Casparian strip integrity, can enhance suberin deposition in wild-type plants^11^ supports our interpretation that enhanced suberin deposition is a response to loss of integrity of the Casparian strip-based apoplastic diffusion barrier. Furthermore, loss-of-function of the receptor-like kinase SGN3, required for sensing of CIFs, blocks the enhanced deposition of suberin in *esb1-1* and *casp1-1casp3-1* based on a chemical analysis of suberin in *esb1-1* (Supplementary Fig. S3), and also on FY 088 staining^4^. We conclude that Casparian strip defects sensed by the CIFs/SGN3 surveillance system lead to enhanced deposition of suberin in the endodermis.

### Enhanced suberin deposition limits solute leakage from the vasculature when Casparian strip integrity is lost

**Figure 2:**
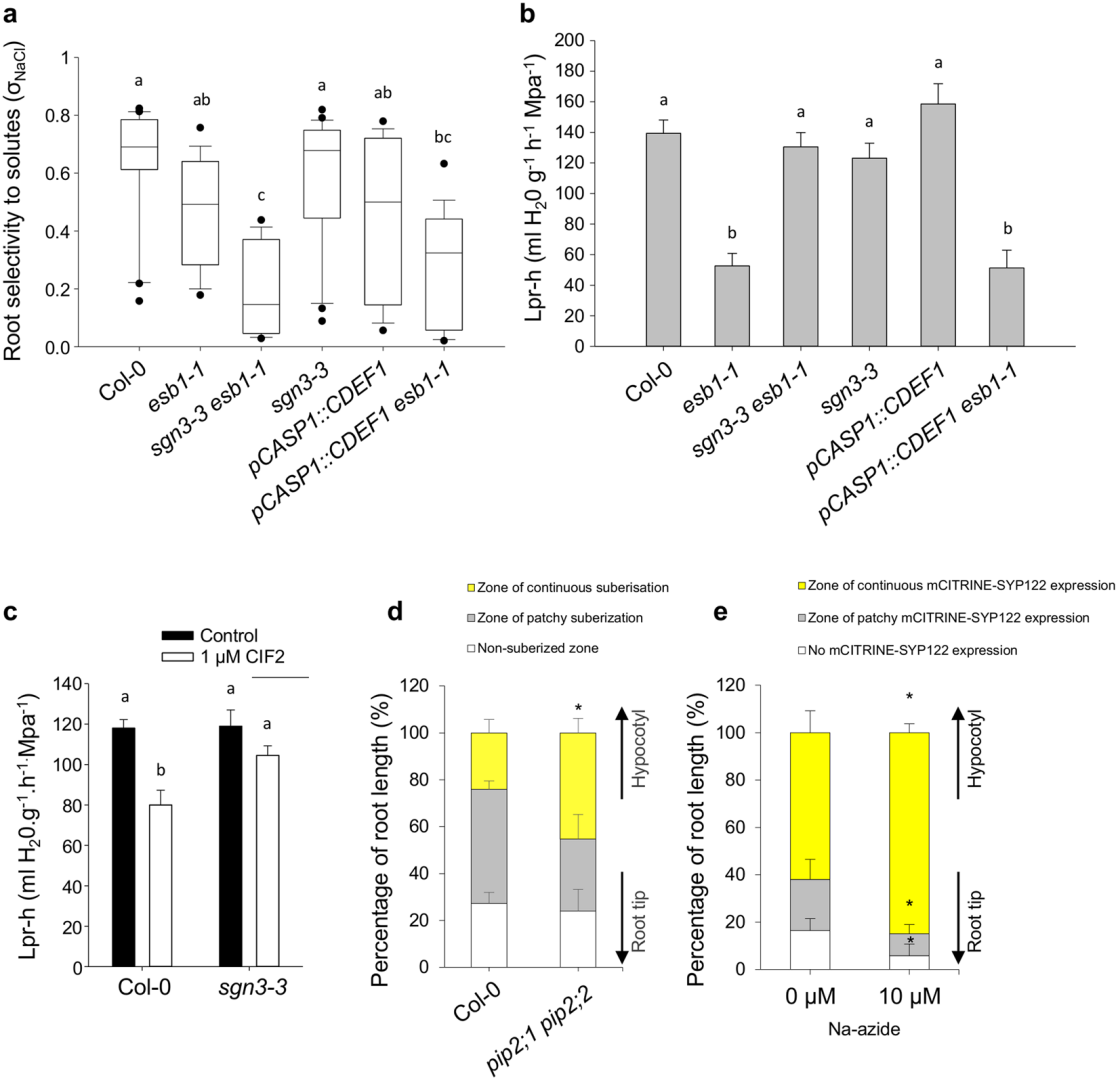
Whole root solute leakage, hydraulic conductivity and apoplastic diffusion barriers. (**a**) Solute leakage into the xylem in 21 day-old plants grown hydroponically subjected to increasing NaCl concentrations. The selectivity of the root to NaCl (σ_NaCl_) was calculated as described in Method section (ANOVA Tukey’s HSD, means ± SE, n = 15–18, pooled from 3 biological replicates, p < 0.05). (**b**) Total root hydraulic conductivity (*L*p_r_) of 21 day-old plants grown hydroponically (ANOVA Tukey’s HSD, means ± SE, n = 18–22, pooled from 3 biological replicates, p < 0.05). (**c**) Effect of the CIF2 peptide and the absence of its receptor (*sgn3-3* mutant) on total root hydraulic conductivity of 21 day-old plants grown hydroponically (ANOVA Tukey’s HSD, means ± SE, n = 8–16, pooled from 3 biological replicates, p < 0.05). (**d**) Effect of the loss of function of the aquaporins *PIP2;1* and *PIP2;2* in the *pip2;1 pip2;2* double mutant on endodermal suberisation. 5 day-old seedlings were stained with Fluorol Yellow 088. Quantification (%) of suberization along the root axis, distinguishing three differentiation stages, non-suberized, patchy and continuous (bottom panel) are presented (t-test, means ± SD, n = 4, p < 0.05). (**e**) Expression pattern of the suberisation reporter line *pGPAT5::mCITRINE-SYP122*. 5 day-old seedlings were treated with 0 and 10 µM sodium azide (NaN_3_) for 6 h. *mCITRINE-SYP122* expression was quantified along the root axis, distinguishing three differentiation stages: continuous, patchy and no expression. Values are expressed as a percentage of root length (t-test, means ± SD, n = 6, p < 0.05).

The observation that enhanced suberin is deposited as a response to loss of integrity of the endodermal-based diffusion barrier between stele and cortex, raises the question, what is the function of this increased suberin deposition? Previously, the extent of endodermal suberin has been shown to be part of the response to nutrient status^6^. We therefore tested the selectivity to solutes σ_NaCl_, in roots varying in the extent of suberin deposition and the functionality of Casparian strips. For this, we measured solute leakage into xylem sap of pressurized roots at increasing sodium chloride concentrations in the solution bathing the roots. Taken individually, σ_NaCl_ of roots of *esb1-1*, *sgn3-3* and wild-type were not significantly different from one another (Fig. 2a), which is surprising given the disruption of the Casparian strip-based apoplastic diffusion barrier in both mutants. However, removal of suberin in *esb1-1*, by endodermal-specific ectopic expression of a cutinase (*CDEF1*, *CUTICLE DESTRUCTING FACTOR 1*) (Supplementary Fig. S3)^7^, caused a significant decrease in σ_NaCl_ compared to wild-type plants (Fig. 2a), and a similar tendency when compared to *esb1-1* (Fig. 2a). This supports the notion that enhanced suberin deposition at the endodermis helps prevent passive solute leakage caused by defects in the Casparian strips of the *esb1-1* mutant. We also observed a significant decrease in σ_NaCl_ in the double mutant *esb1-1sgn3-3* compared to both wild-type and *sgn3-3* (Fig. 2a). It is known that *SGN3* is required for the enhanced deposition of suberin that occurs at the endodermis in *esb1-1*^4^ (Supplementary Fig. S3). Our observation that removal of this enhanced suberin in *esb1-1sgn3-3* decreases σ_NaCl_ further supports our conclusion that the role of this increased suberin deposition is to limit solute leakage where Casparian strip barriers are disrupted.

### Loss of Casparian strip integrity signals an inactivation of aquaporins responsible for reducing root hydraulic conductivity and enhanced suberin deposition

It has also been suggested that endodermal suberin may impact water permeability, though how is still unclear^14^. To further address the role of enhanced endodermal suberin, we investigated root hydraulic conductivity (*L*p_r_) of *esb1-1* and observed a significant reduction by 62% with respect to wild-type (Fig. 2b). Importantly, this difference in *esb1-1 L*p_r_ originates mainly from a reduction in an aquaporin-mediated (sodium azide sensitive) water transport pathway (20.8 ± 4.6 ml.g^−1^.h^−1^.MPa^−1^ in *esb1-1* vs 91.2 ± 4.7 ml.g^−1^.h^−1^.MPa^−1^ for control plants, p < 0.001, Supplementary Fig. S4a). We also observed that the azide-resistant water transport pathway was lower in *esb1-1* than in wild-type (Supplementary Fig. S4a), yet to a lesser extent than the aquaporin mediated pathway. The dramatic reduction in aquaporin-mediated *L*p_r_ in *esb1-1* we observe is an intriguing finding, which led us to consider if this lack of aquaporin activity in *esb1-1* roots is due to a direct output from the CIFs/SGN3 signalling pathway, or if it represents an effect downstream of enhanced suberin deposition. We found that removal of endodermal suberin in *esb1-1* through expression of *CDEF1* in the endodermis had no further effect on *L*p_r_ (Fig. 2b). This rules out a role for suberin in the reduced aquaporin-mediated *L*p_r_ of *esb1-1*. However, in the *esb1-1sgn3-3* double mutant, as compared to *esb1-1*, we observed a full recovery of *L*p_r_ back to wild-type levels (Fig. 2b). Loss of Casparian strip integrity in *esb1-1* therefore appears to be sensed by the CIFs/SGN3 signalling pathway, which leads to the inactivation of aquaporins, thereby reducing *L*p_r_. To support this conclusion, we show that exogenous application of CIF2 to wild-type plants for 3 h induces a reduction in *L*p_r_, and only in the presence of a functional *SGN3* (Fig. 2c).

We have established the existence of two critical outputs of the CIFs/SGN3 diffusion-barrier surveillance system. These are enhanced deposition of endodermal suberin limiting solute leakage, and the inactivation of root aquaporin activity reducing *L*p_r_. Do these two independent outputs of the CIFs/SGN3 diffusion barrier surveillance system work in parallel, or in series with one response leading to the other? The fact that removal of endodermal suberin in *esb1-1* does not compensate for its reduced *L*p_r_ (Fig. 2b) suggests that enhanced suberin deposition is not the cause of the reduced aquaporin-mediated *L*p_r_. However, reduced activity of aquaporins through loss-of-function of the two major aquaporins *PIP2;1* and *PIP2;2* in the *pip2;1pip2;2* double mutant^15^, does cause significant increases in endodermal suberin deposition (Fig. 2d). A similar increase in suberin is also observed after treatment with the aquaporin inhibitor sodium azide through observation of the activity of the transcriptional reporter *pGPAT5::mCITRINE-SYP122* for suberin biosynthesis^6^ (Fig. 2e). *GPAT5* expression is observed to expand toward the root tip after 6 hours only of sodium azide treatment (Fig. 2e). Based on this evidence, we propose the following sequence of events. Casparian strip defects are detected by the apoplastic leakage of CIFs from the stele, being sensed by SGN3. Once activated, SGN3 signals the inactivation of aquaporins thereby reducing *L*p_r_ which in turn leads to the early and enhanced deposition of endodermal suberin. In such a model, SGN3 would inhibit aquaporin function, which may appear at variance with the usual activation of aquaporins through phosphorylation^16^. Yet, such an inhibition was recently described in the case of FERONIA, a protein kinase inactivating PIP2; 1 function through an as yet unknown mechanism^17^.

### Activation of endodermal ABA signalling is not required for enhanced suberin deposition in responses to loss of Casparian strip integrity

**Figure 3:**
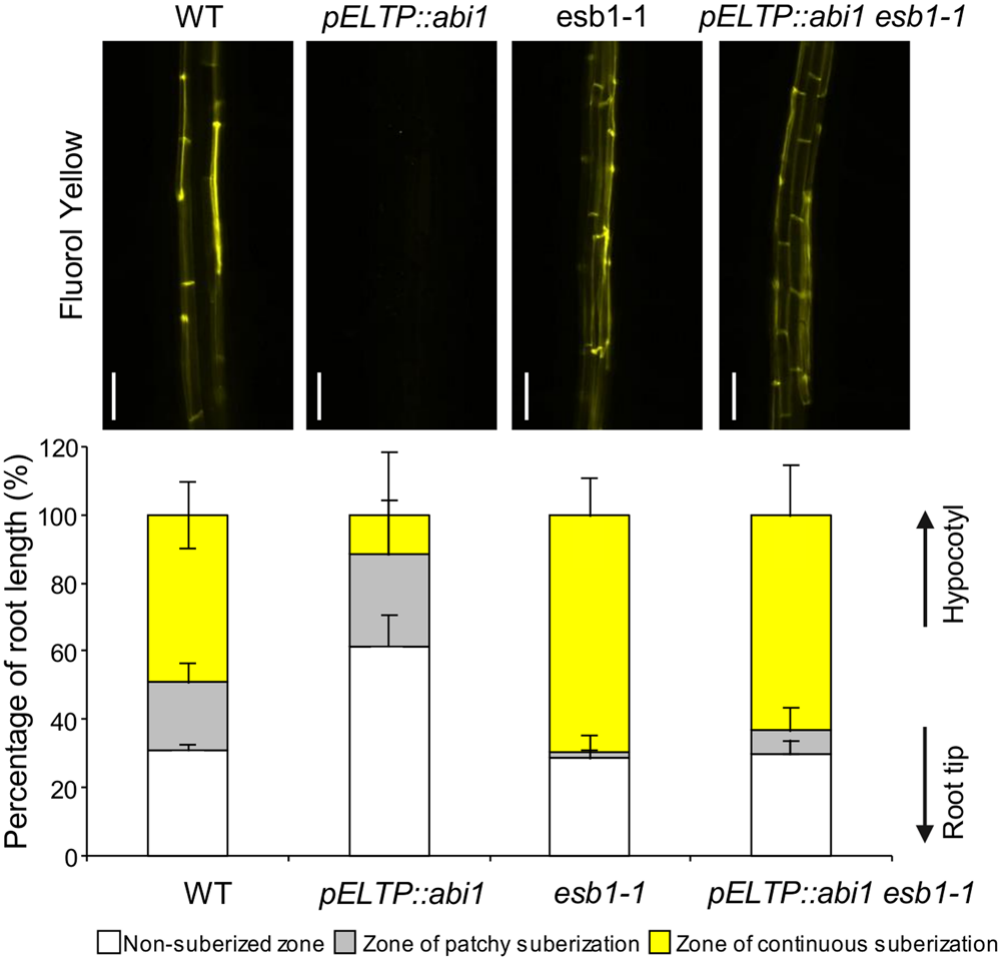
Activation of ABA signalling at the endodermis is not involved in the enhanced deposition of suberin. Fluorol Yellow 088 staining for suberin in 5 day-old WT, *pELTP::abi1-1*, *esb1-1* and *pELTP::abi1-1esb1-1* roots. Pictures taken in similar parts of the root (upper panels) and quantification (%) of suberization along the root axis, distinguishing three differentiation stages, non-suberized, patchy and continuous (bottom panel) are presented (means ± SD, n ≥ 10). Scale bar, 50 μm.

Abscisic acid (ABA) has been shown to be involved in regulating both aquaporin activity reviewed in^18^ and suberin deposition^6^, making ABA an interesting candidate worth exploring for a role in downstream CIFs/SGN3 signalling. To probe this possibility we expressed the dominant negative allele of the regulator of ABA signalling *ABA-INSENSITIVE 1* (*ABI1*) in the endodermis of *esb1-1* using *pELTP::abi1*. This *abi1* construct specifically blocks ABA signalling at the endodermis and delays suberisation in a wild-type background (Fig. 3 and Supplementary Fig. S4a) as previously shown in (6). In *esb1-1*, we observed *abi1* to have no effect on either the inactivation of aquaporins or the enhanced deposition suberin (Fig. 3 and Supplementary Fig. S4a). We also observe that aquaporin inhibition with sodium azide in the *pELTP::abi1-1* line still induces expression of the suberin biosynthesis gene *GPAT5* toward the root tip in the *pGPAT::mCITRINE-SYP122* line, as observed in wild-type (Supplementary Fig. S4b, Fig. 2e). Based on this, activation of ABA signalling in the endodermis does not link perception of Casparian strip defects with the downstream responses of reduced aquaporin-mediated *L*p_r_ or suberin deposition. Supporting this, we observe no significant difference in ABA concentration in roots of *esb1-1* and wild-type (Fig. S4c). Interestingly, suppression of endodermal ABA signalling seems to contribute to the inactivation of aquaporin-mediated *L*p_r_ in a wild-type background (Supplementary Fig. S4a).

### A root-based SGN3 derived signal mediates stomata closures by ABA locally in the leaves

The *esb1-1* mutant is known to have reduced stomatal apertures and enhanced wilting resistance^3,10^. This suggests that the CIFs/SGN3 sensing system not only initiates a local root response to Casparian strip integrity but is also involved in initiating long-distance responses in the shoot. We observe reduced stomatal apertures in *esb1-1* (Supplementary Fig. S5a), and an analysis of the expression of a set of known ABA signalling and response genes in leaves (Supplementary Fig. S5b–i) suggest that this stomatal closure is part of an ABA driven response. The *aba1* mutation confers a strong ABA deficiency^19^. By generating an *esb1-1aba1* double mutant, we investigated the ABA-dependent component in the leaf response we observe in *esb1-1*. ABA-deficiency in *esb1-1aba1* suppressed both the reduced stomatal aperture (Supplementary Fig. S6a) and the activation of expression of ABA signalling and response genes (assayed as expression of *RAB18*, *RD29B*, *CER1*) that we observe in *esb1-1* (Supplementary Fig. S6b–d). ABA is therefore necessary for the stomatal closure we observe in *esb1-1*. The elevated ABA concentration we observe in leaves of *esb1-1* compared to wild-type (Supplementary Fig. S4d) supports this conclusion. We also used the *esb1-1sgn3-3* double mutant to test if *SGN3* is involved in initiating this leaf ABA response. In leaves of the *esb1-1sgn3-3* double mutant the elevated expression of a set of ABA signalling and response genes observed in *esb1-1* is reduced to below that of wild-type (Supplementary Fig. S5b–i). Further, the reduced stomatal aperture of *esb1-1* is also recovered to wild-type levels in this double mutant (Supplementary Fig. S5a). *SGN3* is therefore necessary for the ABA-dependent stomatal closure in response to the defective endodermal diffusion barrier in *esb1-1*.

This raises the question of what links detection of a break in the endodermal diffusion barrier with ABA-driven closure of stomates in the leaf? Removal of endodermal suberin in *esb1-1* expressing *CDEF1* revealed a significant reduction in ABA-regulated gene expression, and a tendency to increasing stomatal aperture towards wild-type (Supplementary Fig. S5). Thus, increased suberin deposition in the endodermis of the *esb1-1* root appears to play a partial role in the ABA controlled reduction in leaf transpiration. We have ruled out a role of local ABA signalling in controlling enhanced suberin deposition at the endodermis in *esb1-1* (Fig. 3). Using a similar strategy of expressing *abi1* in the endodermis, in this case using the *SCARECROW* promoter (*pSCR*), primarily active in the endodermis^20^, we also show that in *esb1-1* ABA signalling at the endodermis is not promoting stomatal closure (Supplementary Fig. S6e) or enhanced ABA signalling in leaves (assayed using expression of *RAB18*, *RD29B* and *CER1)* (Supplementary Fig. S6f–h). We note that *pSCR* is also active in bundle sheath cell^21^, and so ABA-signalling in these cells is also not involved in promoting stomatal closure in *esb1-1*.

Furthermore, enhanced ABA signalling in the endodermis is also not responsible for the initiation of the long-distance response of stomatal closure in leaves, and again it is more likely that suppression of ABA signalling is playing a role. This can be seen in the fact that expression of *abi1* in the endodermis, blocking ABA signalling, mimics the effect of *esb1-1* on *L*p_r_ and stomatal aperture closure (Supplementary Figs S4a and S6e). However, these possibilities remain to be further explored. In contrast to these root-based or long-distance effects, the closure of stomata in leaves in response to a root-based CIFs/SGN3 derived signal is mediated by ABA locally in the leaves. We also note that the long distance signal connecting CIFs/SGN3 in roots with reduced leaf transpiration is currently unknown. Interestingly, a root-derived peptide (CLE25) has been recently identified as involved in long-distance signalling^22^. In response to drought stress, CLE25 move from root to shoot and induces ABA accumulation in leaves and stomatal closure.

### Output of CIFs/SGN3 surveillance compensates for loss of diffusion barrier function

**Figure 4:**
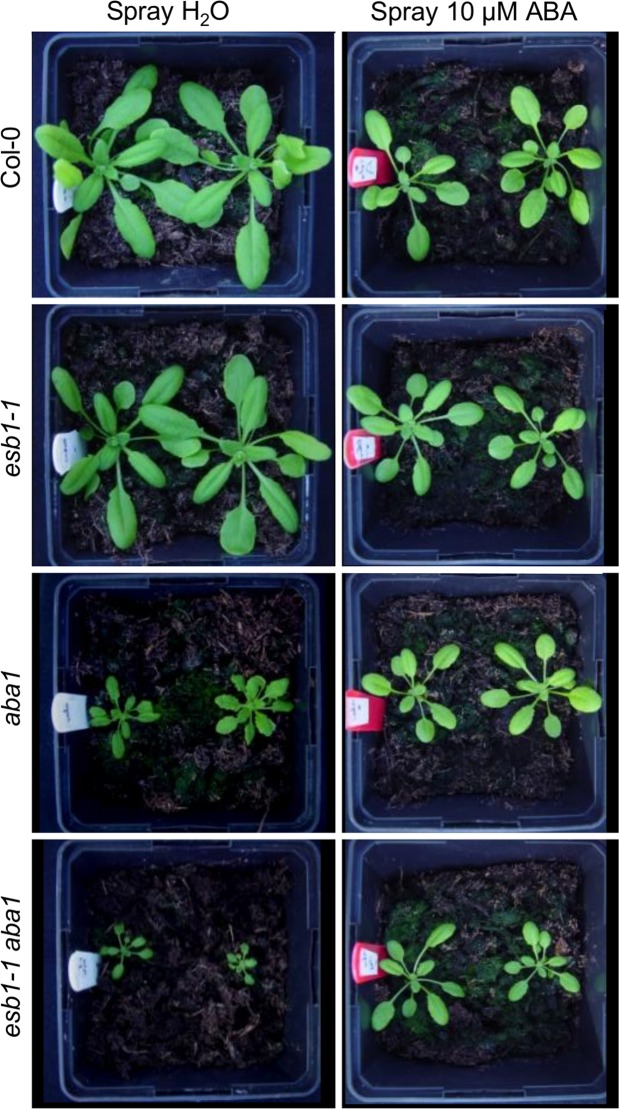
ABA biosynthesis is essential for physiological compensation of the *esb1* defect. Phenotype of Col-0, *esb1-1*, *aba1* and *esb1-1aba1* plants grown 26 days in the greenhouse at the early reproductive stage. The plants were sprayed with water or with 10 µM ABA.

Casparian strips have been suggested to play a critical role in forming a barrier to apoplastic diffusion to limit uncontrolled uptake and backflow of solutes from roots reviewed in^1^. However, most Casparian strip mutants only appear to show fairly subtle phenotypic effects, and this has been a source of continued puzzlement. Here, we show that sensing damage to Casparian strips via leakage of the vasculature-derived CIF peptides from the stele into the cortex triggers a mechanism that inactivates aquaporins, promotes enhanced deposition of suberin limiting solute leakage in roots, and reduces transpiration in leaves, which all contribute to increasing solute concentration in the xylem (Supplementary Fig. S7). The overall outcome of this integrated response is a rebalancing of solute and water uptake and leakage. These physiological compensation mechanisms mitigate the loss of Casparian strip integrity, allowing relatively normal growth and development. A key part of this compensation mechanism is the ability of *esb1-1* to limit water loss by the shoot by reducing stomatal aperture, in an ABA-dependent manner. This is clearly established by our observation that the *esb1-1aba1* double mutant has severely reduced growth and seed production compared to either of the single mutants, and these growth defects can be partially supressed by the exogenous application of ABA (Fig. 4, Supplementary Figs S8 and S9).

The mechanisms we have identified are triggered by the loss of Casparian strips integrity. Such an event can occur during biotic stress including root nematodes infestation, and also during developmental processes such as lateral root emergence (LRE) where Casparian strips are remodelled^23^, suberin deposition occurs^7^, and aquaporin expression is suppressed^15^.

**Figure 5:**
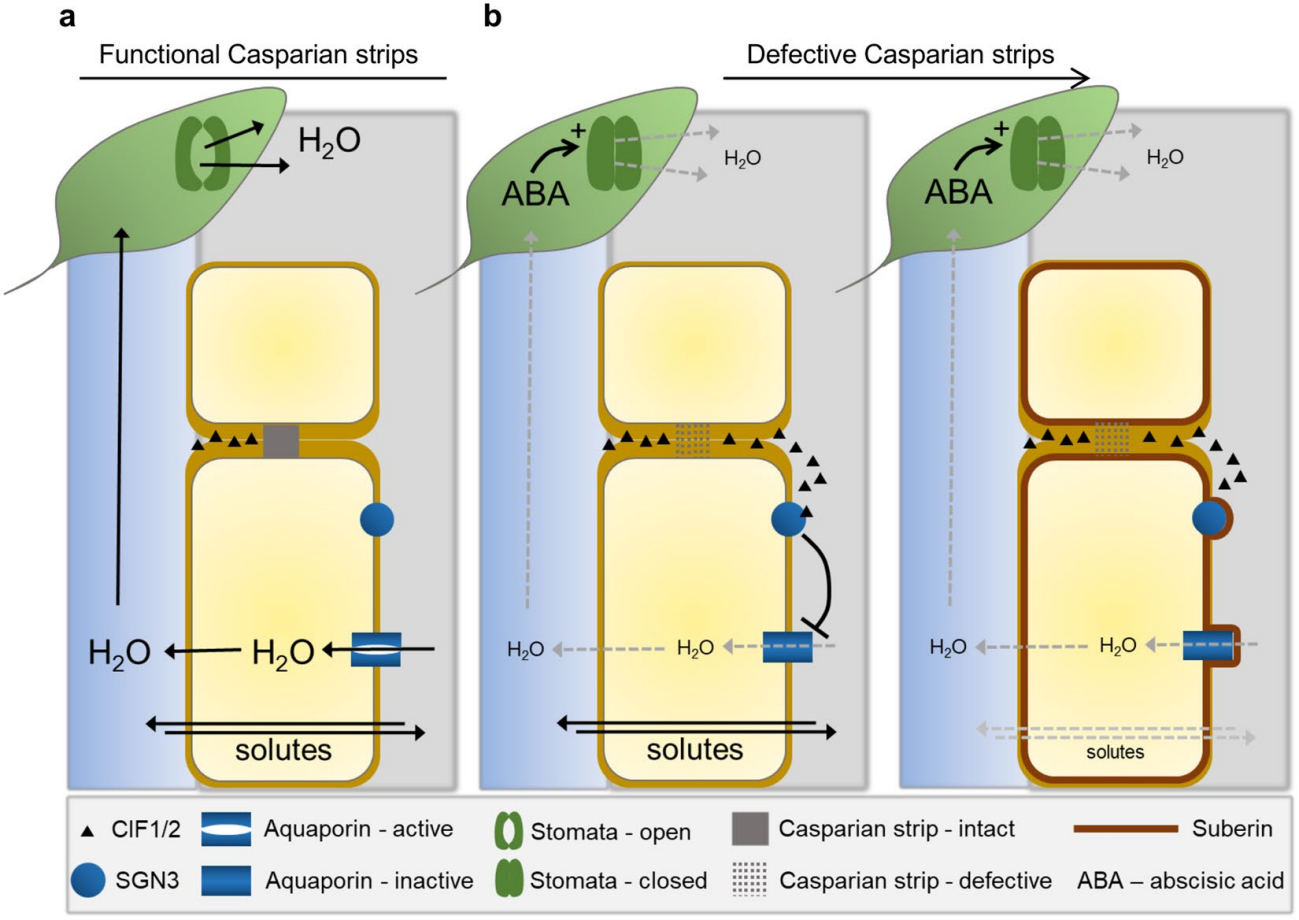
Model summarising the integration between apoplastic endodermal diffusion barriers, hydraulic conductivity, solute permeability and stomatal conductance. (**a**) Functioning Casparian strips at the endodermis prevent the apoplastically localised peptide CIF1&2 from diffusing from the stele, across the endodermis, and into the cortex. (**b**) Defective Casparian strips are detected by leakage of CIF1&2 into the cortical apoplast where the peptides are sensed by binding to SGN3, signalling inactivation of aquaporins. This leads to reduced hydraulic conductivity and closure of stomates in leaves through a process mediated locally by ABA. Inactivation of aquaporins leads to the enhanced deposition of suberin which reduces solute conductivity across the root into and out of the xylem.

Here, we describe novel outputs of the CIFs/SGN3 surveillance system that couple sensing of the integrity of the Casparian strip-based apoplastic diffusion barrier at the endodermis with pathways that regulate both solute leakage and hydraulic conductivity in the root (Fig. 5). Long distance signals then connect these root-based responses with compensatory mechanisms in leaves which are mediated by local ABA signalling (Fig. 5). Our discoveries provide a new framework which integrates our emerging understanding of the molecular development of the Casparian strip and suberin diffusion barriers with two of the major physiological functions required for plant survival – solute and water uptake.

## Methods

The *esb1-1* mutant was induced by fast neutron bombardment in a Col-0 background^3^, *aba1* (N25407) generated by T-DNA insertion was provided by the Nottingham Arabidopsis Stock Centre (NASC). Marker lines for monitoring the suberin in the root of young seedlings including *pFAR1::GUS*, *pFAR4::GUS*, *pFAR5::GUS*, *pCASP1::GFP* and *pGPAT5::NLS-GFP* seeds with Col-0 background were provided by Domergue Frédéric^24^ and Niko Geldner^6,25^. To confirm the homozygous nature of the mutations in crosses between *esb1-1* and the suberin reporter lines above, genotyping was done by PCR or sequencing. The seeds of *sgn3-3*, *esb1-1sgn3-3*, *pCASP1::CDEF1* and *esb1-1 pCASP1::CDEF1* for root hydraulic and suberin monomers measurement have been described in the references^4,6^, and were provided by Niko Geldner and Takehiro Kamiya^7^. The seeds of *pELTP::abi1*, *pELTP::mCitrine-SYP122* and *pSCR::abi1* was provided by Marie Barberon and José R. Dinneny. The *pip2;1pip2;2* mutant was provided by Christophe Maurel^15^. The *pELTP::*abi1 line was crossed with *pELTP::mCitrine-SYP122* and the T1 were analysed for mCitrine-SYP122.

### Grafting of Arabidopsis

For grafting experiment, we used the protocol described in^26^. Arabidopsis seeds were sterilized and sowed in plates containing ½ MS, 1% Agar, and 0.5% sucrose. After 2 days of stratification at 4 °C, plants were grown in short-day conditions for one week (10 h. light/14 h dark) Then, the seedlings were cut as described in^26^. Shoots and roots were grafted in a new plate with the same medium and grown for 10 days. Then, adventitious roots emerging at or above the junction were removed from subsequent analyses.

### Optical microscopy

Autofluorescence and FY 088 were detected with standard GFP filter with excitation at 488 nm and 500–600 nm band path filter by using a Zeiss LSM700 confocal microscopy^3,27,28^. Briefly, for observation of Casparian strips lignin with basic fuchsin and CASP1-GFP, 2.5 days old seedlings were grown on plates contatining ½ MS, 1% Agar. Seedlings were cleared and stained as described^29^. Suberin observation was done by FY 088 staining as described^28^. GUS-staining for observation of the early appearance of the expression of suberin biosynthesis gene was done as described^25^, and photos were taken by using stereo microscopes connected with a digital camera. For mounting root on glass slides, 50% glycerol was used. Quantification of the number of cells after the onset of elongation was done as described^25^. 2.5 days old plant were counted from the day when plates were taken out from the 4 °C and put into the growth chamber, with the 16 h. (light): 8 h. (dark) long-day conditions was set. Expression patterns of the suberisation reporter line pGPAT5::mCITRINE-SYP122 were observed using an epifluorescence microscope with GFP filter as described for FY 088 staining^6^. The length of the root and the length of the continuous suberisation zone were measured with the ImageJ software.

### Real time quantitative RT-PCR analysis

qRT-PCR was done for the analysis of the transcript level of suberin biosynthesis genes and ABA-related genes in wild-type Col-0, *esb1-1*, *esb1-1pCASP1::CDEF1*, *pCASP1::CDEF1*, *sgn3-3*, *esb1-1sgn3-3*, *pSCR::abi1*, *pSCR::abi1esb1-1*, *aba1*, and *aba1esb1-1*. Two reactions were done per biological sample and 4 independent replicate samples per genotype were used to evaluate the transcript abundance of each genotype. Total RNA of leaves was extracted using TRIzol reagent (Invitrogen) and purified using RNeasy MinElute Cleanup Kit (Qiagen). After DNase treatment (Invitrogen), an equal amount of total RNA (1.5 μg) was used for RT with anchored Oligo(dT) 12–18 primer (Invitrogen). SuperScript®II reverse transcriptase was used for obtaining the first strand cDNA. FastStart Universal SYBR Green Master (ROX) (Roche) was used for Real time qRT-PCR. CT values were determined based on the efficiency of amplification and the mean CT values were normalized against the corresponding Ubiquitin10 gene (AT4G05320), and the expression of per genotype finally was calculated using the 2^(−DDCT)^ method^30^.

### Stomatal aperture measurements

Fully expanded leaves from 6–7 weeks old plant were grown in the greenhouse or growth chamber (80–100 μmol m^−2^ s^−1^ light intensity, a short-day photoperiod 10 h. light/14 h. dark). Leaves were harvested 3 to 5 h after lights were on. To determine stomatal aperture, we modified the protocol described in^31^. Nail polish was applied to the abaxial surface of leaves, when fully hardened a piece of transparent scotch tape was used to get out the film of clear nail polish from the surface of the leaf, the tape with the stomatal impression in the nail polish was placed onto a slide and observed under a light microscope (Zeiss Axioskop 40) at 20x objective and images were recorded digitally by ProgRes® CapturePro 2.9 Image Capture Software. Data were collected by measuring the maximum inner width of the stomatal pore from the captured photographs of a minimum of 50 stomata from 5 individual leaves per genotype and condition. Three independent measurements were done for the comparison between Col-0 and *esb1-1*, two independent measurements were done for the other mutant lines.

### Exogenous ABA treatments

Seeds were sowed on the surface of the compost pots. After 2 days stratification in the 4 °C cold room, all the pots were transferred into the greenhouse with a controlled environment (250–300 μmol m^−2^ s^−1^ light intensity, a long-day photoperiod 16 h. light/8 h dark, 19 to 22 °C). Each pot contains two plants of the same genotype, and each genotype of Col-0, *esb1-1*, *aba1* and *aba1esb1-1* has four pots (N = 8 for each genotype were grown). About 100 mL of 10 µM exogenous ABA (Sigma, A1049) was sprayed onto the leaves of plants (Col-0, *esb1-1*, *aba1* and *aba1esb1-1*) twice a week from the 8th day counted from the day when pots were transferred into the greenhouse from the 4 °C cold room. The control group was sprayed with the same amount of Milli-Q water. The treatment was continued until the 50th day, when the number of the siliques was quantified. Plants were bottom-watered twice per week with 0.25 × Hoagland solution.

Growth conditions for root hydraulic measurements and determination of ABA concentration. Seeds were surface sterilized and sowed into clear polystyrene culture plates (12 × 12 cm) containing ½ MS adjusted to pH 5.7. The plates were kept 2d at 4 °C and then incubated vertically for 10d under environmentally-controlled conditions: 60% relative humidity, 16 h/d of 250 µmol photons m^−2^ s^−1^ (long days) and 20 °C. Plants were later transferred into a hydroponic growth system consisting in a 35 × 35 cm plastic floating base over a basin filled with 8 L of hydroponic solution: 1.25 mM KNO_3_, 0.75 mM MgSO_4_, 1.5 mM, Ca(NO_3_)_2_, 0.5 mM KH_2_PO_4_, 50 mMFeEDTA, 50 mM H_3_BO_3_, 12 mM MnSO_4_, 0.70 mM CuSO_4_, 1 mM ZnSO_4_, 0.24 mM MoO_4_Na_2_, and 100 mM Na_2_SiO_3_. The different physiological determinations were done after 10–11 days of hydroponic culture.

### Determination of plant water relations

Root hydrostatic conductance (*K*_r_) was determined in freshly detopped roots using a set of pressure chambers filled with hydroponic culture medium^32^. Excised roots were sealed using dental paste (Coltène/Whaledent s.a.r.l., France) and were subjected to 350 kPa for 10 min to achieve flow stabilization, followed by successive measurements at pressures 320, 160, and 240 kPa. Root hydrostatic conductance (K_r_) was calculated by the slope of the flow (J_v_) to pressure relationship. The hydrostatic water conductivity (*L*p_r−h,_ ml H_2_O g^−1^ h^−1^ MPa^−1^) was calculated by dividing *K*_r_ by the root dry weight. For sodium azide (NaN_3_) experiments, *L*p_r−h_ was determined from continuous Jv measurement at 320 kPa as described^33^. The effect of the CIF2 peptide on the *L*p_r−h_ of Col-0 and *sgn3-3* mutant was determined by subjecting the plants to 1 µM concentration of CIF2 (11) for 3 h, and afterwards determining its *L*p_r−h_ by using 320, 160, and 240 kPa in pressure chambers as explained before.

### Selectivity to solutes

The passive leakage of solutes, and subsequent selectivity (σ_NaCl_), of the root was tested by determining the osmotic potential of the xylem sap in plants under constant pressure of 300 kPa in the pressure chamber under a series of NaCl solutions from 0 to 150 mM. The osmotic potential of the sap was measured using a Vapro 5520 osmometer (Wescor, USA) and was plotted against the osmotic potential of each salt treatment concentration and its slope represented selectivity of the root to NaCl, or σ_NaCl_.

### Xylem sap osmotic potential

The plants were de-topped with a razor blade at the root – rosette junction and the isolated hypocotyl was immediately introduced into a 100 µl micro capillary. Dental paste (Coltène/Whaledent s.a.r.l., France) was used to ensure a proper seal between the hypocotyl and the capillary. We left the plants exuding for 10 min and later on the xylem sap was collected after another 45 min and its osmolality was measured using a Vapro 5520 osmometer (Wescor, USA).

### Suberin Chemical Analysis

For quantitative chemical analysis suberin was extracted, depolymerised, derivatised and quantified as by GC-MS as previously described^10^.

### ABA quantification

Extraction and purification of indole-3-acetic acid (IAA) and abscisic acid (ABA) metabolites was done as described previously^34^ with minor modifications. Frozen samples were homogenized using a MixerMill (Retsch GmbH, Haan, Germany) and extracted in 1 ml 50 mM sodium phosphate buffer (pH 7.0) containing 1% sodium diethyldithiocarbamate and stable isotope-labelled internal standards (5 pmol of [^13^C_6_]-IAA and [^6^H_2_]-ABA per sample added). The pH was adjusted to 2.7 with 1 M hydrochloric acid, and the samples were purified by solid phase extraction. The extracts were purified on Oasis HLB columns (30 mg, Waters Corp., Milford, USA), conditioned with 1 ml methanol, 1 ml water, and 0.5 ml sodium phosphate buffer (pH 2.7). After sample application, the column was washed with 2 ml 5% methanol and then eluted with 2 ml 80% methanol. Eluates were evaporated to dryness and dissolved in 30 ul of mobile phase prior to mass analysis using a 1290 Infinity Binary LC System coupled to the 6490 Triple Quad LC/MS System with Jet Stream and Dual Ion Funnel technologies (Agilent Technologies)^35^.

### Statistical Analyses

The data were analysed using either analysis of variance (ANOVA) with the MIXED procedure or by using pair t-test in SAS (version 9.2, SAS Institute Inc., NC, USA). For ANOVA analyses, Tukey’s honest significant difference (HSD) or Duncan’s multiple range test (MRT) post-hoc adjustments were used to determine significant differences between treatments means at α = 0.05.

## Supplementary information

Supplemental Information
