# Supplementary material for: Surveillance of cell wall diffusion barrier integrity modulates water and solute transport in plants
Source: Sci Rep. 2019 Mar 12;9:4227. doi: 10.1038/s41598-019-40588-5 (PMC6414709; doi:10.1038/s41598-019-40588-5)
Supplement: Supplementary file 1 — Supplemental Information [file 41598_2019_40588_MOESM1_ESM.pdf]

Surveillance of cell wall diffusion barrier integrity modulates water and solute transport in plants.

Peng Wang[1,8#], Monica Calvo-Polanco[2,#], Guilhem Reyt[3], Marie Barberon[4,9], Chloe Champeyroux[2], Véronique Santoni[2], Christophe Maurel[2], Rochus B Franke[5], Karin Ljung[6], Ondrej Novak[6,7] , Niko Geldner[4], Yann Boursiac[2] and David E Salt[*1,3]

[1]Institute of Biological and Environmental Sciences, University of Aberdeen, Aberdeen AB24 3UU, UK.

[2]Biochimie & Physiologie Moléculaire des Plantes, Univ Montpellier, CNRS, INRA, SupAgro, Montpellier, France

[3]Current address: Division of Plant and Crop Sciences, School of Biosciences, University of Nottingham, LE12 5RD, UK

[4]Department of Plant Molecular Biology, University of Lausanne, 1015 Lausanne, Switzerland.

[5]Department of Ecophysiology, Institute of Cellular and Molecular Botany, University of Bonn, 53115 Bonn, Germany

[6]Umeå Plant Science Centre, Department of Forest Genetics and Plant Physiology, Swedish University of Agricultural Sciences, SE-901 83 Umeå, Sweden

[7]Laboratory of Growth Regulators, Centre of the Region Haná for Biotechnological and Agricultural Research, Faculty of Science of Palacký University & Institute of Experimental Botany of the Czech Academy of Sciences, Olomouc, Czech Republic.

[8]Current address: Department of Agronomy and Horticulture, University of Nebraska Lincoln, Lincoln, NE 68588-0660, USA.

[9]Current address: Department of Botany and Plant Biology, University of Geneva, 30, quai Ernest-Ansermet, CH-1211 Geneva 4, Switzerland.

[#]These authors contributed equally to the work.

[*]**Corresponding author**:
David E Salt
Division of Plant and Crop Sciences, School of Biosciences,
University of Nottingham, LE12 5RD, UK
david.salt@nottingham.ac.uk
Phone #: +44 (0) 115 9516332

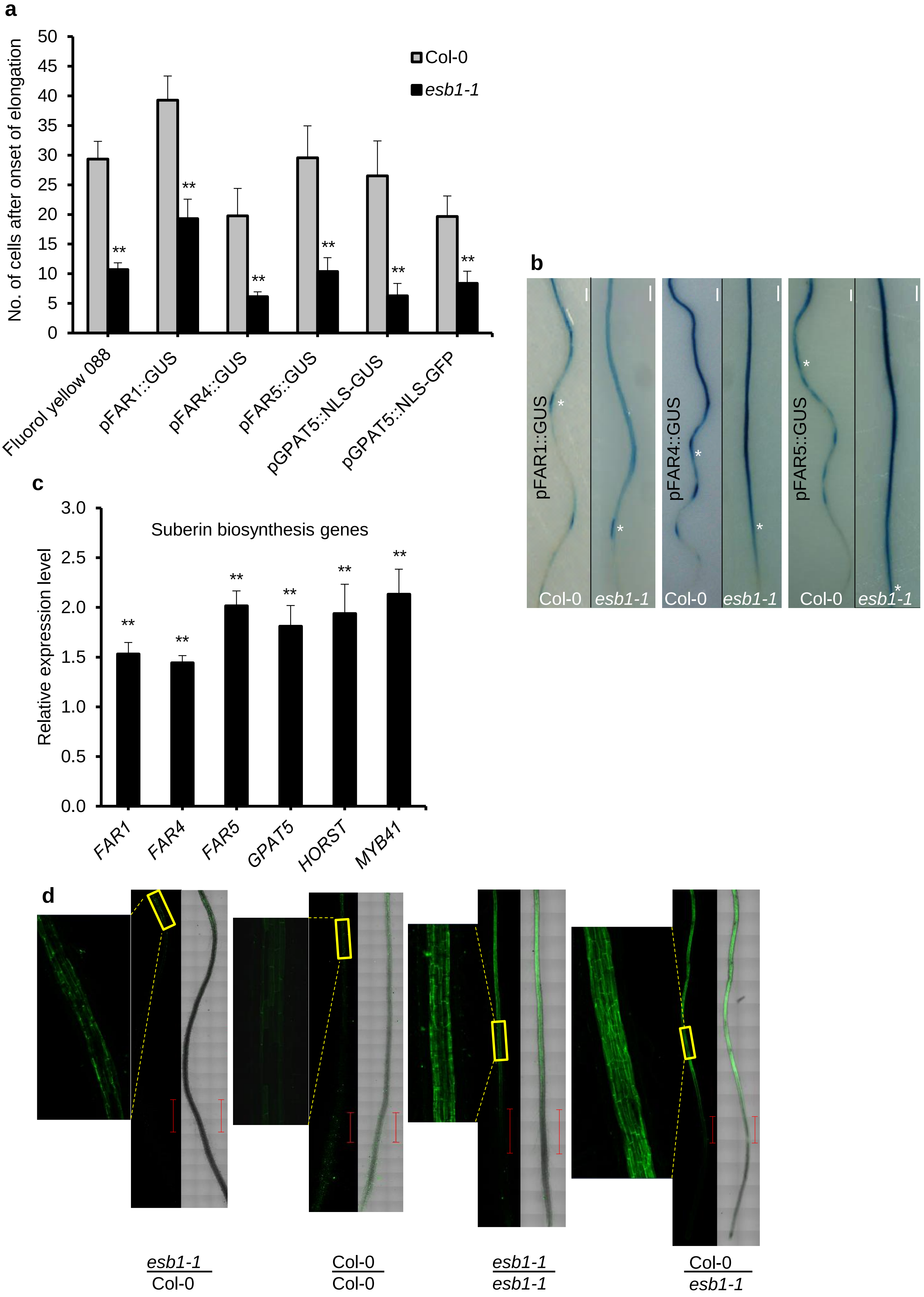

Supplemental Figure 1. **Enhanced endodermal suberin in *esb1-1* is driven by loss-of-function of *ESB1* in roots.**

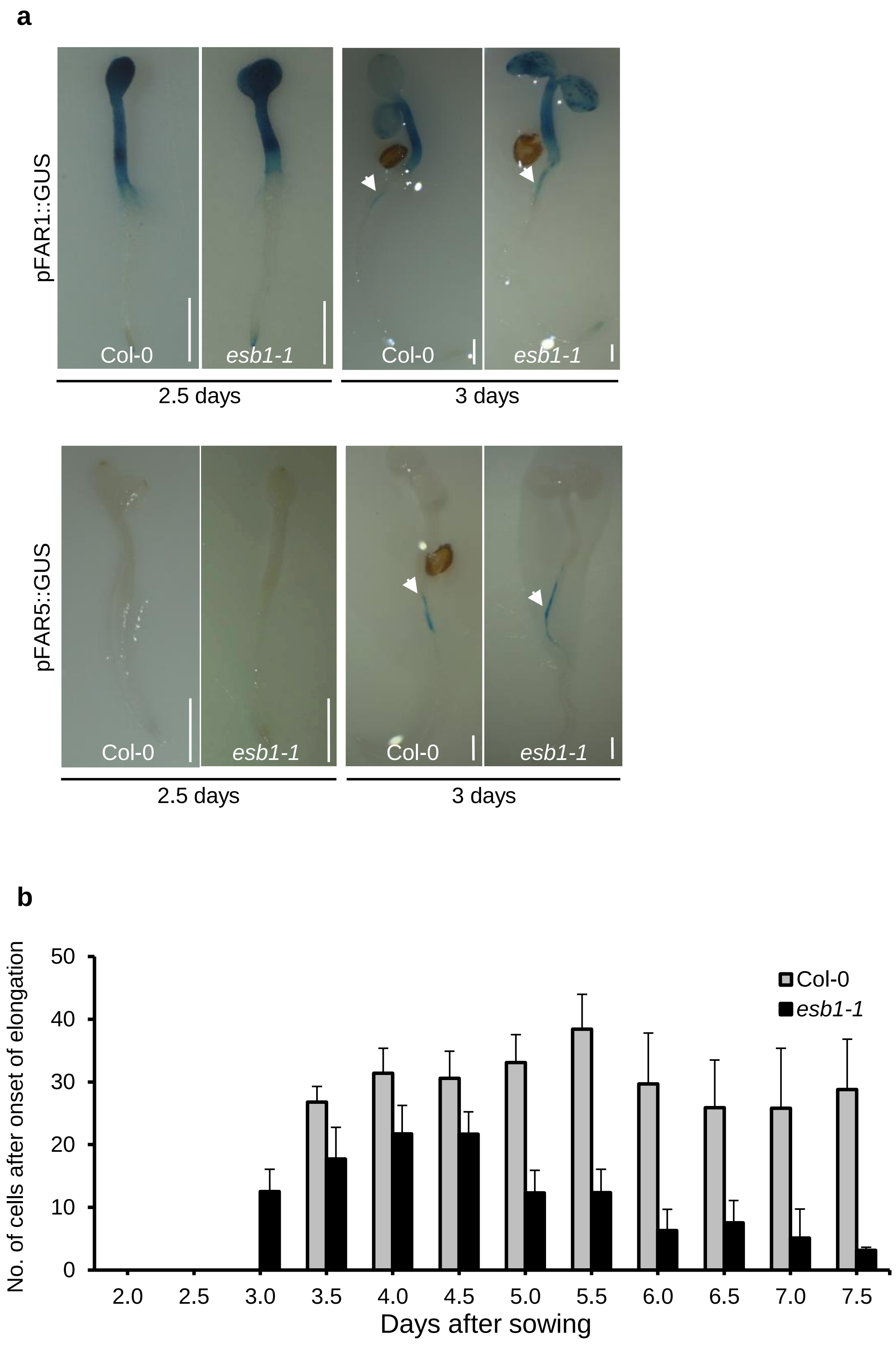

Supplemental Figure 2. **Casparian strip defects in *esb1-1* precede enhanced endodermal suberin deposition.**

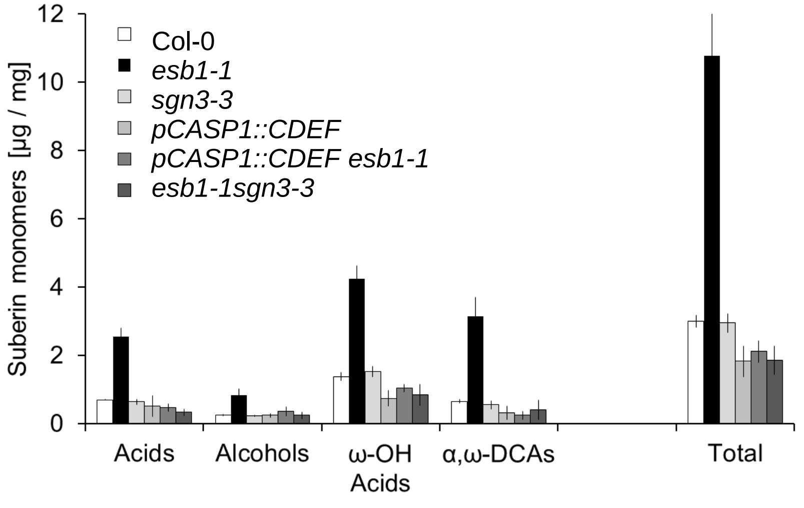

Supplemental Figure 3. **Endodermal suberin content.**

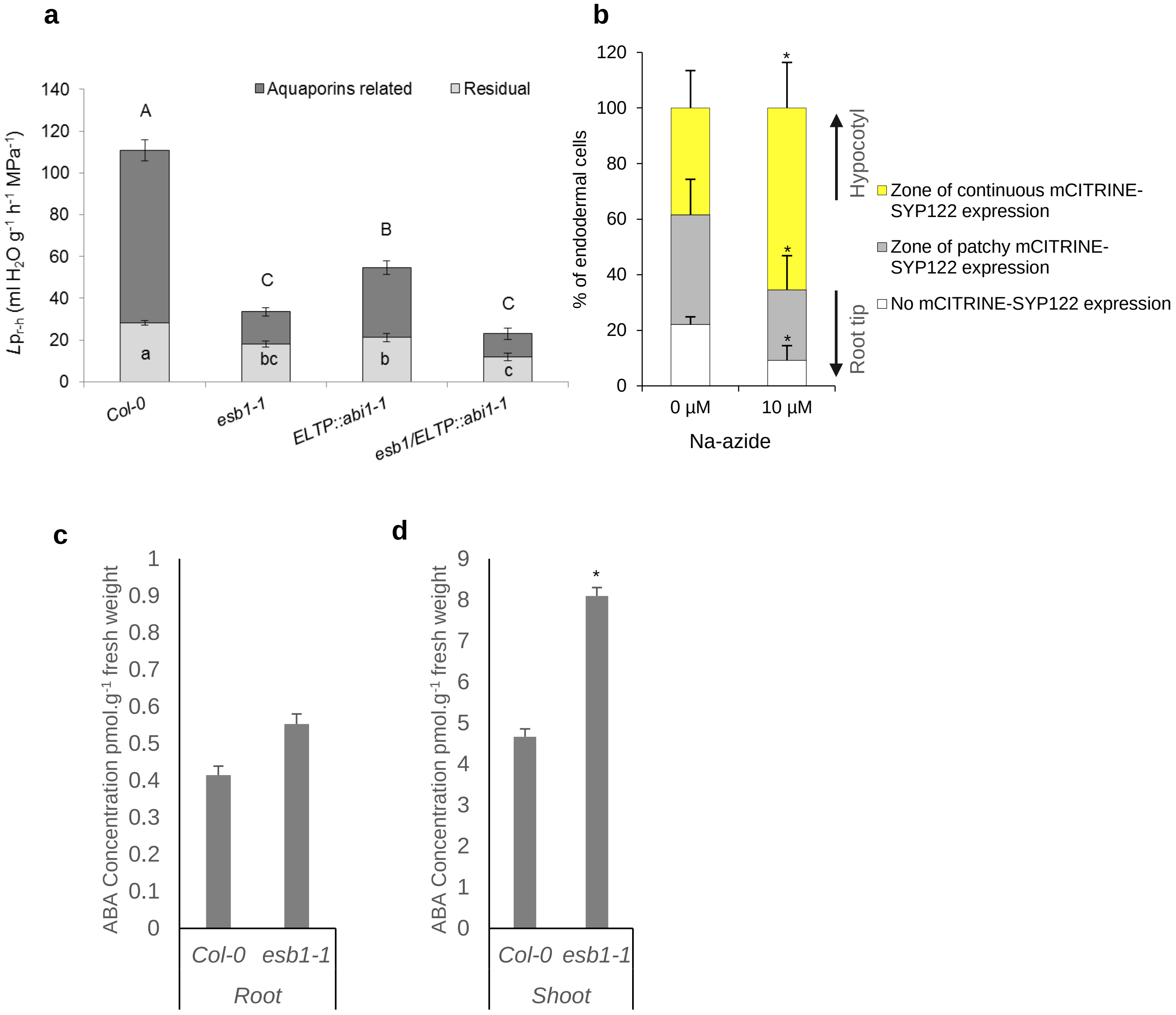

Supplemental Figure 4. **Activation of ABA signalling at the endodermis is not involved in the enhanced deposition of suberin or the inactivation of aquaporin-mediated hydraulic conductivity**.

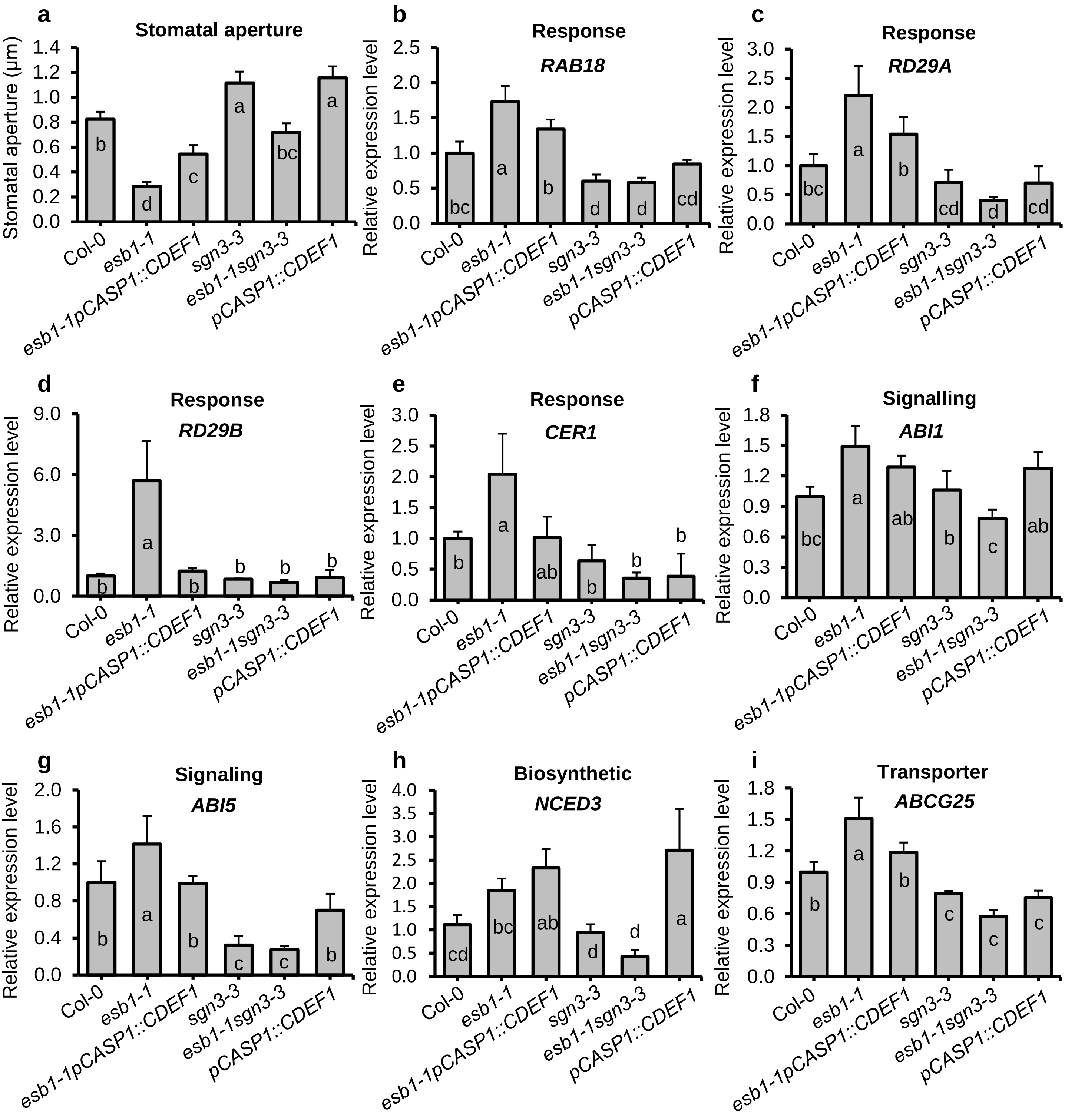

Supplemental Figure 5. **Casparian strip defects in *esb1-1* is associated with a SGN3-dependent reduction of the stomatal aperture and an increased expression of ABA-related genes in leaves.**

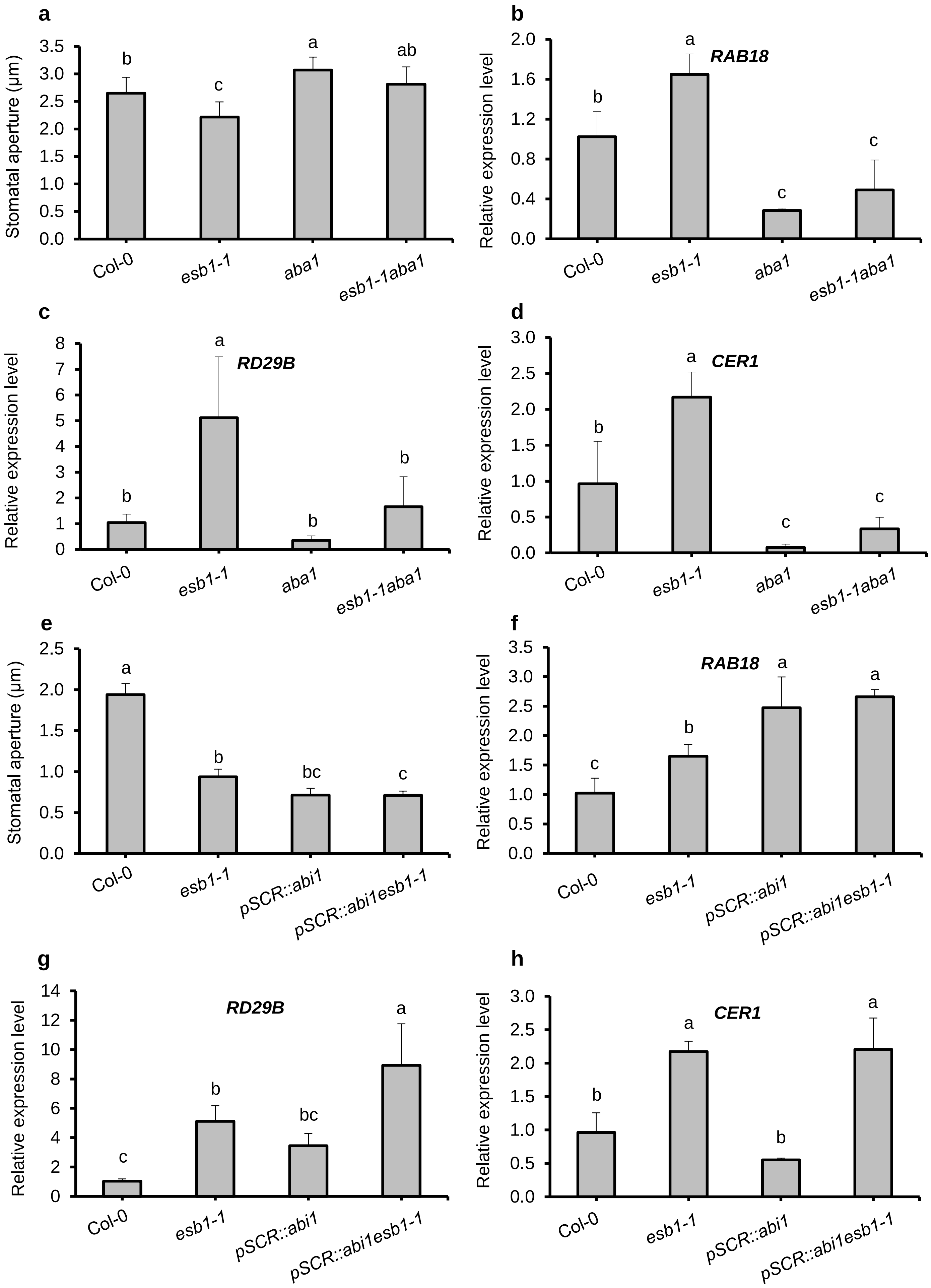

Supplemental Figure 6. **ABA signalling is necessary for the stomatal closure in leave.**

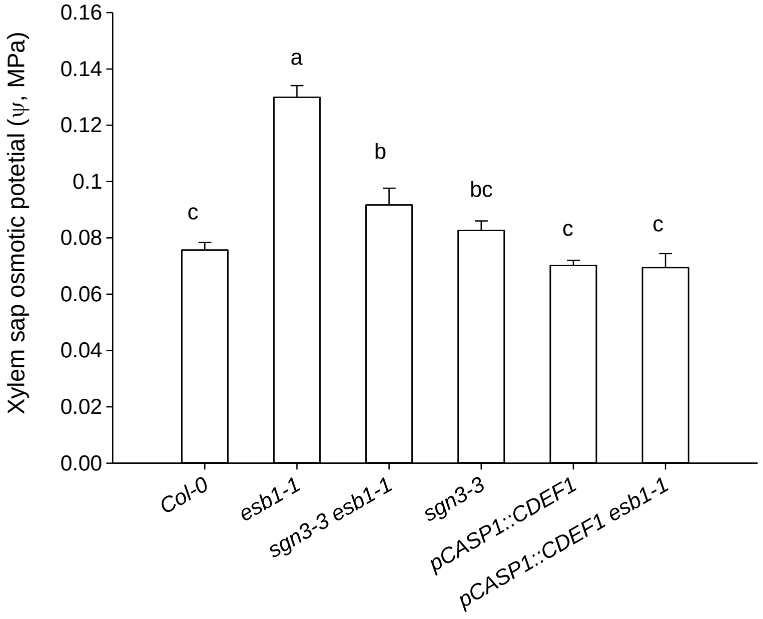

Supplemental Figure 7. **Impact of apoplastic barriers on xylem solute concentration.**

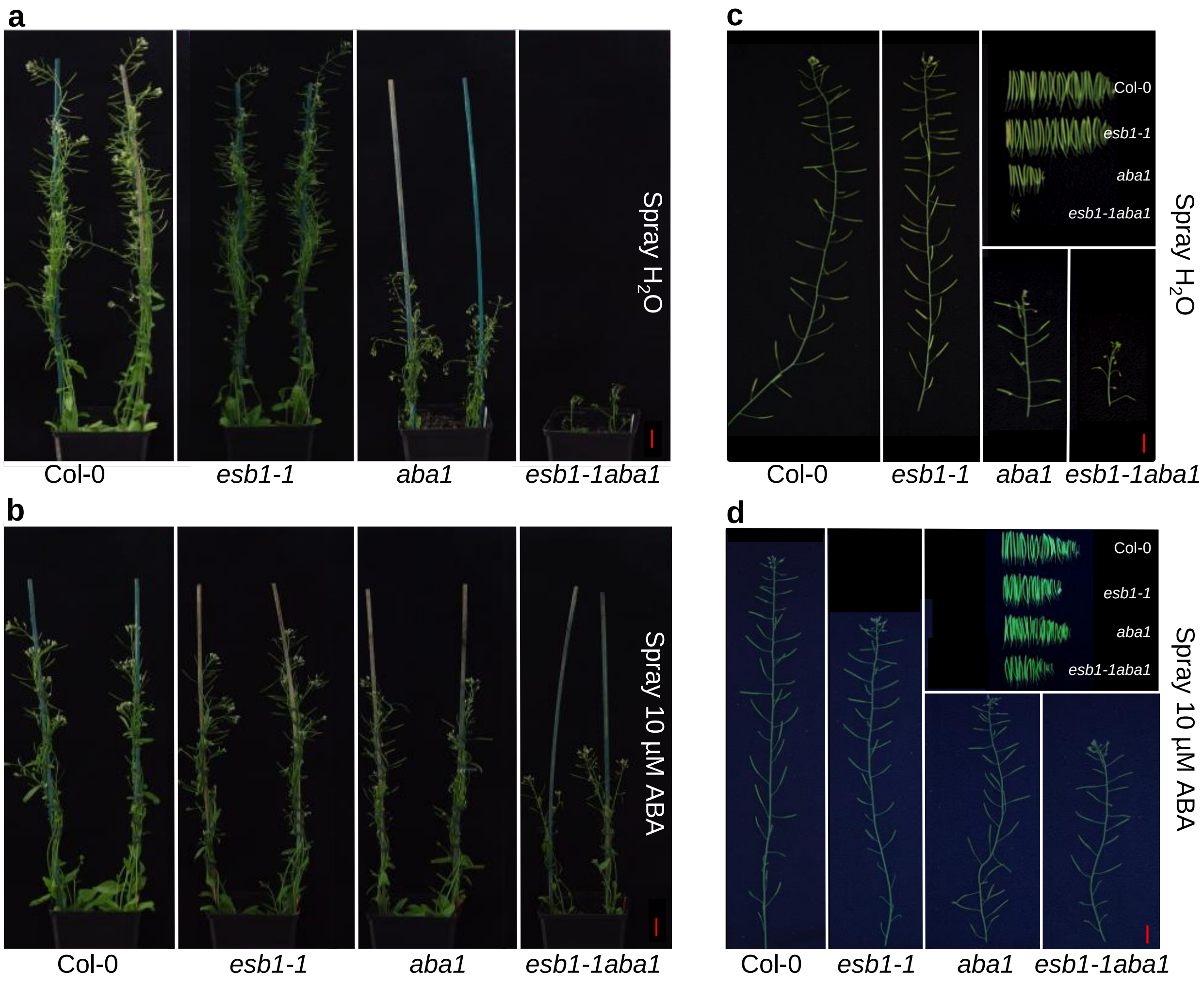

Supplemental Figure 8. **ABA biosynthesis is essential for physiological compensation of the *esb1-1* defect**

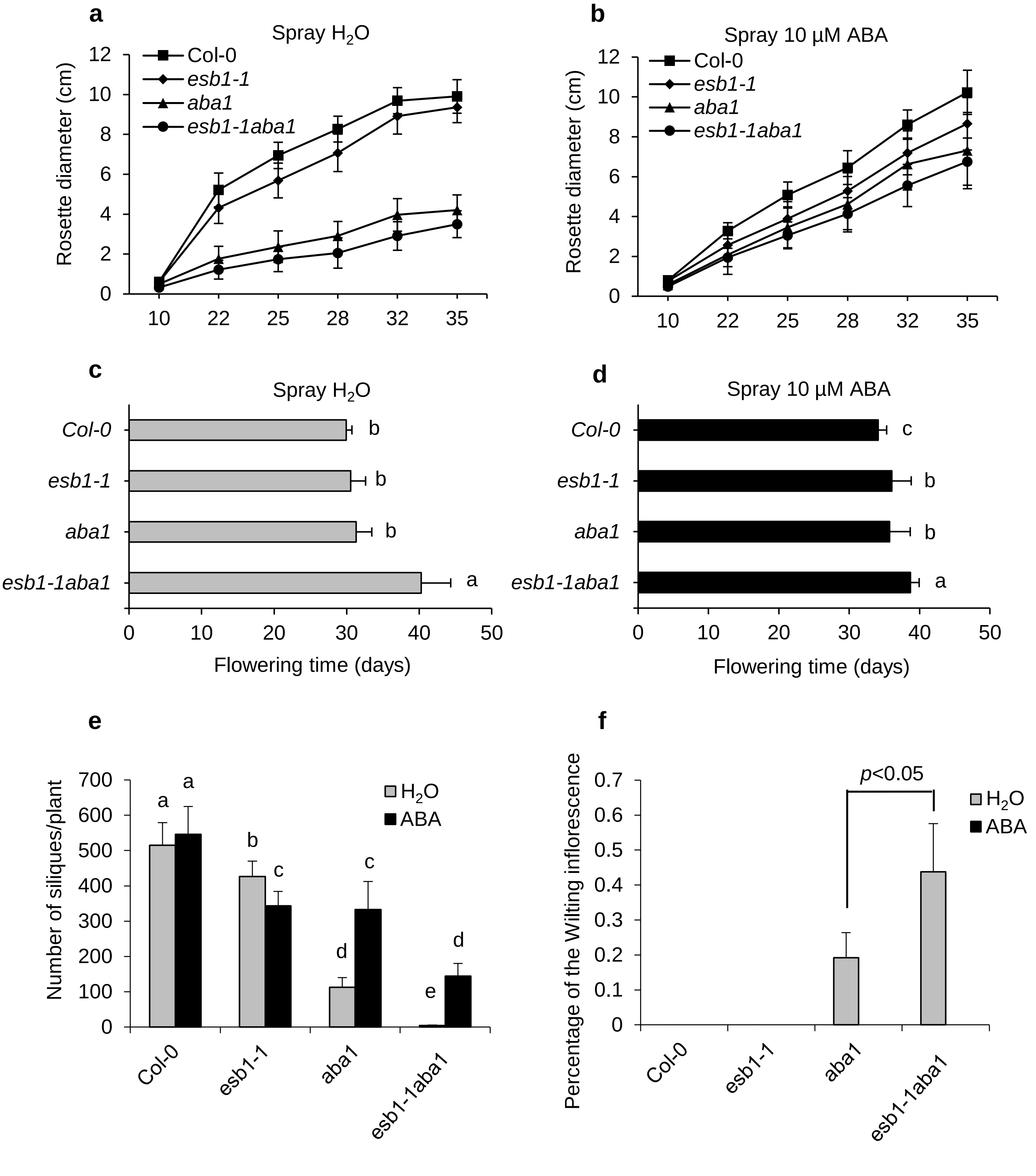

Supplemental Figure 9**. ABA biosynthesis is essential for physiological compensation of the *esb1-1* defect.**
